# Electrically pumped soliton microcombs on thin-film lithium niobate

**DOI:** 10.1515/nanoph-2025-0510

**Published:** 2025-12-11

**Authors:** Xiaomin Lv, Ze Wang, Tianyu Xu, Chen Yang, Xing Jin, Binbin Nie, Du Qian, Yanwu Liu, Kaixuan Zhu, Bo Ni, Qihuang Gong, Fang Bo, Qi-Fan Yang

**Affiliations:** State Key Laboratory for Artificial Microstructure and Mesoscopic Physics and Frontiers Science Center for Nano-Optoelectronics, School of Physics, Peking University, Beijing 100871, China; Hefei National Laboratory, Hefei 230088, China; Collaborative Innovation Center of Extreme Optics, Shanxi University, Taiyuan 030006, China; Peking University Yangtze Delta Institute of Optoelectronics, Nantong 226010, China; Nankai University, Tianjin 300071, China

**Keywords:** thin-film lithium niobate, microcombs, integrated photonics

## Abstract

Thin-film lithium niobate (TFLN) has enabled efficient on-chip electro-optic modulation and frequency conversion for information processing and precision measurement. Extending these capabilities with optical frequency combs unlocks massively parallel operations and coherent optical-to-microwave transduction, which are achievable in TFLN microresonators via Kerr microcombs. However, fully integrated Kerr microcombs directly driven by semiconductor lasers remain elusive, which has delayed integration of these technologies. Here, we demonstrate electrically pumped TFLN Kerr microcombs without optical amplification. With optimized laser-to-chip coupling and optical quality factors, we generate soliton microcombs at a 200 GHz repetition frequency with an optical span of 180 nm using only 25 mW of pump power. Moreover, self-injection locking enables turnkey initiation and substantially narrows the laser linewidth. Our work provides integrated comb sources for TFLN-based communicational, computational, and metrological applications.

## 1 Introduction

Lithium niobate (LN) is renowned for its strong second-order nonlinearities, enabling electro-optic modulation, second-harmonic generation, and spontaneous parametric down-conversion. The advent of thin-film lithium niobate (TFLN) has translated these capabilities to photonic chips by supporting low-loss LN integrated circuits [1], [2]. State-of-the-art devices now include high-speed, low-drive-voltage electro-optic (EO) modulators [3], [4] and high-efficiency periodically poled LN (PPLN) waveguides that extend toward the ultraviolet [5], [6]. These components are rapidly transitioning to commercial use across many fields.

Optical frequency combs (OFCs) comprise evenly spaced spectral lines that, under Fourier transform, correspond to periodic pulse trains [7]. Beyond tabletop mode-locked lasers, chip-scale realizations have emerged in high-*Q* microresonators, where resonant field enhancement drives nonlinear frequency generation [8]. Two principal mechanisms are employed: electro-optic modulation and Kerr nonlinearity. In electro-optic combs, the line spacing is set by the microwave drive (typically <100GHz ), whereas Kerr microcombs offer broader flexibility with repetition frequency determined by the resonator free-spectral range (FSR) and, owing to relaxed phase-matching constraints, can access wider optical bandwidths. These attributes position Kerr microcombs for applications in parallel communications [9], [10], optical computing [11], [12], [13], and optical clocks [14].

On TFLN, Kerr microcombs have typically required amplified continuous-wave pumps to reach the parametric oscillation threshold, hindering full integration [15], [16], [17], [18]. By contrast, in centrosymmetric, ultralow-loss platforms such as Si_3_N_4_, direct semiconductor-laser pumped microcombs have been demonstrated and deployed [19], [20]. Realizing fully integrated TFLN Kerr microcombs has thus hinged on mitigating losses – specifically, improving laser-to-chip coupling and achieving sufficiently high *Q* to enable parametric oscillation at modest pump powers.

**Figure 1:**
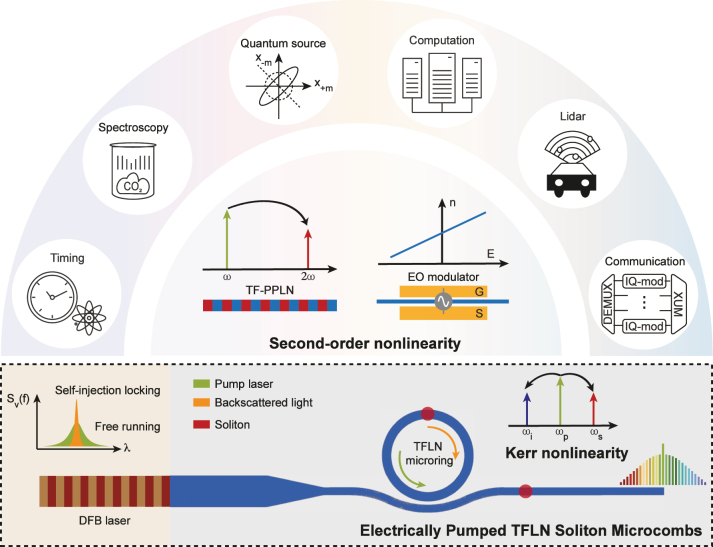
Electrically pumped TFLN soliton microcombs and representative applications. The integration of electrically pumped soliton microcombs with other second-order nonlinear optical components on TFLN creates a versatile platform for a broad range of applications, including optical clocks, spectroscopy, quantum information processing, optical computing, LiDAR, and communications.

Here, we demonstrate integrated TFLN Kerr microcombs directly driven by a distributed-feedback (DFB) laser (Figure 1). Engineered edge couplers deliver approximately 25 mW of on-chip pump power, sufficient to initiate parametric oscillation in a microresonator with *Q* ≈ 3 × 10^6^. The operation is robust owing to self-injection locking (SIL), which provides turnkey initiation and narrows the laser linewidth to approximately 4.7 kHz. The resulting soliton microcombs feature a 200 GHz repetition frequency and an optical span exceeding 180 nm.

## 2 Methods

### 2.1 Device design

**Figure 2:**
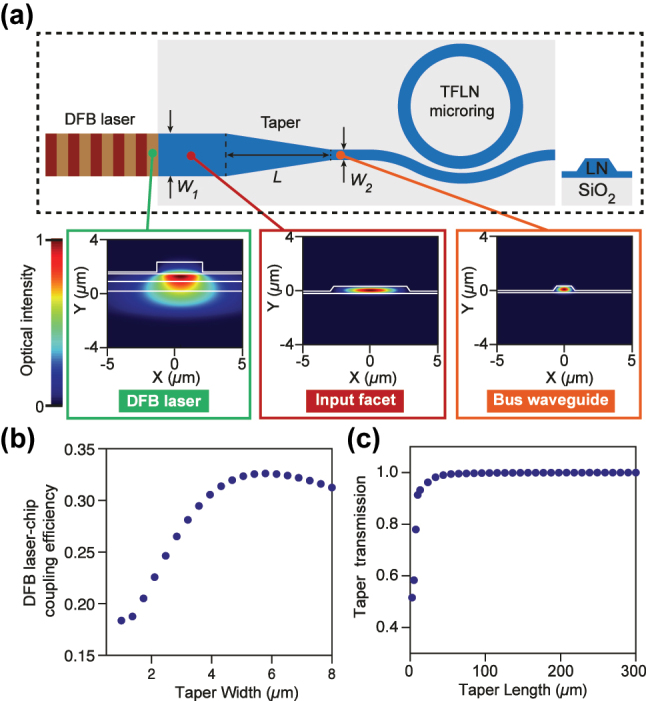
Device layout. (a) The DFB laser and the microresonator. The LN waveguide width is reduced from *W*_1_ to *W*_2_ over a length *L* to enable efficient coupling to the laser. Insets: simulated fundamental transverse-electric (TE) modes of the DFB laser (left), the input facet (middle), and the bus waveguide (right). (b) Simulated laser-chip coupling efficiency as a function of the facet size. (c) Simulated transition efficiency as a function of the taper length.

The detailed device configuration is shown in Figure 2a. The DFB laser (DC-1550-HP-02 V008, Shijia) emits a near-Gaussian mode with a large mode-field diameter owing to weak index confinement. The microresonator is fabricated on a *z*-cut LN on-insulator wafer with an LN layer thickness of 600 nm, partially etched by 390 nm, with a wedge angle of approximately 60°, forming a ridge width of 1.2 μm. To mitigate photorefraction, the device is air-clad [21]. Because the mode of the bus waveguide (1 μm wide) is severely mismatched to the laser mode, a taper is introduced: the waveguide width is expanded at the facet and then transitions adiabatically to the narrow bus waveguide. Numerical simulations predict an optimal laser-to-chip coupling efficiency of 33 % at *W*_1_ = 5.8 μm (Figure 2b). To suppress multimode excitation while maintaining high efficiency, we select *W*_1_ = 3.3 μm, which yields a weaker higher-order mode content and an acceptable coupling efficiency of 28 %. Adiabatic behavior is achieved to set the transition length *L* ≳ 100 μm (Figure 2c); in practice, we choose *L* = 300 μm.

Given the DFB laser output power of ≈170mW at 500 mA, the maximum available on-chip pump power is <50mW . This budget imposes a stringent requirement on the parametric oscillation threshold *P*_th_: empirically, comb initiation is reliable when the pump exceeds (3–4) × *P*_th_. We, therefore, set *P*_th_ ≤ 12.5 mW as a design target.

The parametric oscillation threshold is given by [22]

(1) $$P_{\text{th}}=\frac{\pi n\;\omega_{0}A_{\text{eff}}}{4n_{2}}\frac{1}{\eta {\left(1-\eta \right)}^{2}}\frac{1}{D_{1}Q_{\text{o}}^{2}},$$

where *Q*_o_ is the intrinsic quality factor, *n* = 2.21 is the TFLN refractive index, *ω*_0_/2*π* = 193 THz is the pump frequency, *A*_eff_ = 0.765 μm^2^ is the effective mode area, *n*_2_ = 1.8 × 10^−19^ m^2^/W is the Kerr nonlinear index, and *D*_1_/2*π* is the FSR. The loading factor *η* describes the portion of coupling losses in total loss; to minimize *P*_th_, we found *η* = 1/3 is the optimal condition.

**Figure 3:**
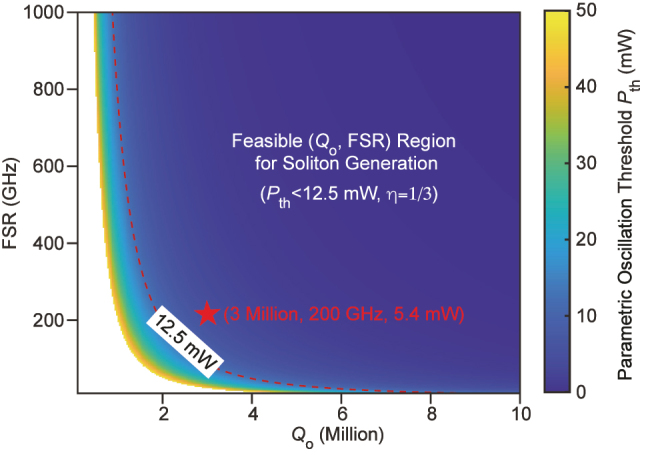
Design optimization for DFB-laser–pumped parametric oscillation. Threshold power map *P*_th_ as a function of intrinsic quality factor *Q*_o_ and FSR under the assumption of optimal coupling *η* = 1/3. The blue region indicates the soliton generation space, bounded by the red dashed line at *P*_th_ = 12.5 mW. The star marker denotes the target operating point (*Q*_o_ = 3 × 10^6^, FSR = 200 GHz) with a calculated threshold of 5.4 mW.

Figure 3 presents the calculated threshold for the TFLN microresonator as a function of *Q*_o_ and FSR under optimal coupling. Increasing *Q*_o_ significantly reduces *P*_th_ and broadens the accessible range of FSRs for a fixed pump budget, whereas lower *Q*_o_ necessitates larger FSRs to maintain low thresholds. For our typical fabrication performance (*Q*_o_ ≈ 3 × 10^6^; see Section 3.1), the analysis indicates that FSR ≳ 200 GHz enables robust soliton generation within the 50 mW budget. We thus chose 200-GHz FSR for the microresonator.

### 2.2 Device fabrication

We employed an optimized fabrication process to realize high-*Q* resonators on commercial *z*-cut TFLN on insulator chips (NANOLN) [23]. The devices were patterned by electron beam lithography using an 800 nm-thick hydrogen silsesquioxane (HSQ) resist. A multipass writing technique was used to ensure high-quality pattern definition. The patterns were subsequently etched by argon-ion (Ar^+^)-based inductively coupled plasma (ICP). After etching, the residual HSQ was removed using diluted hydrofluoric acid. Deposited residues were eliminated by performing two consecutive cleanings in Standard Clean 1 (SC-1) solution (a mixture of NH_4_OH, H_2_O_2_, and H_2_O) heated to ∼85°C . Finally, bus waveguide facets were exposed by manual cleaving.

## 3 Results

### 3.1 Device characterization

**Figure 4:**
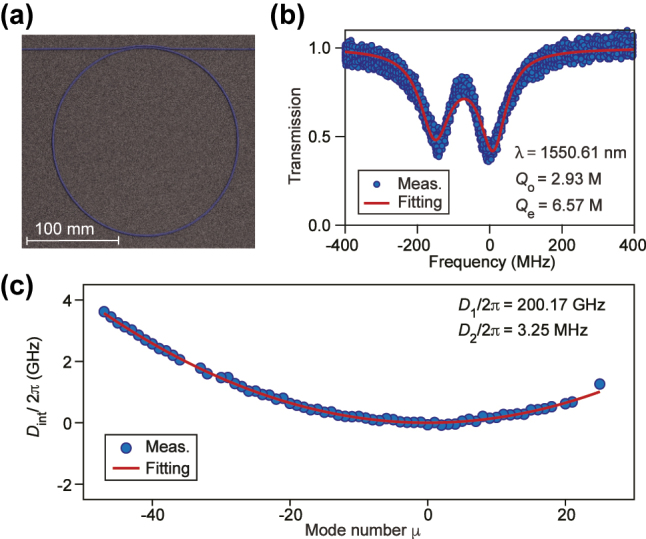
Device characterization. (a) Scanning electron micrograph of the microresonator. (b) Normalized transmission near *λ* = 1,550.61 nm with a fit yielding *Q*_o_ = 2.93 × 10^6^ and *Q*_e_ = 6.57 × 10^6^ (blue: data; red: fit). (c) Integrated dispersion *D*_int_/2*π* of the fundamental TE mode.

To characterize the fabricated resonator (Figure 4a), we record the transmission spectra using a tunable external-cavity diode laser whose frequency sweep is calibrated by an unbalanced Mach–Zehnder interferometer. The resonance doublet observed in Figure 4b arises from Rayleigh backscattering that couples counter-propagating modes [24]. Fitting the doublet lineshape yields an intrinsic quality factor *Q*_o_ = 2.93 × 10^6^ and a coupling quality factor *Q*_e_ = 6.57 × 10^6^. From these, the loading factor is *η* ≈ 0.3, corresponding to an estimated parametric oscillation threshold of *P*_th_ = 5.4 mW for the parameters used in our design model.

We next expand the laser scan to extract the dispersion profile of the fundamental TE family over 1,510–1,630 nm. The integrated dispersion is defined as $D_{\text{int}}=\omega_{\mu }-\omega_{0}-D_{1}\mu =\sum_{n\geq 2}\frac{D_{n}\mu^{n}}{n!},$ where *ω*_*μ*_ is the resonant frequency of the *μ*th mode relative to the pumped mode. As shown in Figure 4c, we obtain FSR = *D*_1_/2*π* = 200.17 GHz and, from a polynomial fit to *D*_int_, a second-order dispersion *D*_2_/2*π* = 3.25 MHz. This indicates anomalous dispersion that is essential for achieving mode-locking in the form of bright solitons in the microresonator.

To quantify the insertion loss at the laser–chip interface, we first measure the lensed-fiber–to–chip-facet coupling via a fiber–chip–fiber transmission experiment with the laser detuned from cavity resonances. Comparing the measured fiber output with the inferred on-chip power yields a laser-to-chip coupling efficiency of ∼20 % (insertion loss ≈7dB ), modestly lower than the simulated 28 % (≈5.5dB) . We attribute the discrepancy to fabrication tolerances, including facet roughness and slight sidewall tilt introduced during dicing and cleaving (see Supplementary Materials).

### 3.2 Comb generation

**Figure 5:**
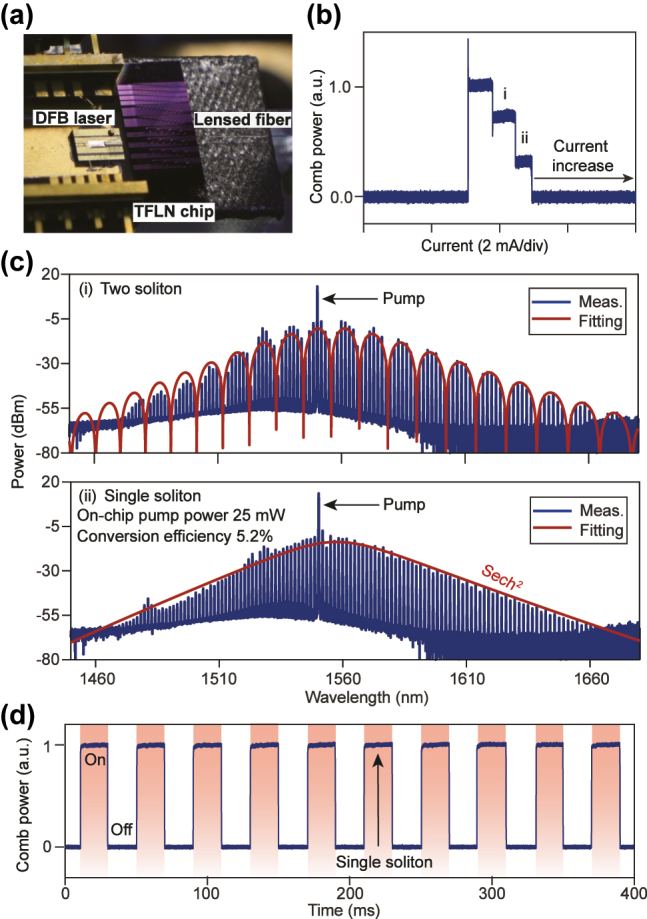
Experimental soliton microcomb generation. (a) Experimental setup. (b) Measured comb power versus driving current of the DFB laser. (c) Optical spectra for (i) a two-soliton state and (ii) a single-soliton state, corresponding to the plateaus in panel (b). (d) Repeatable turnkey initiation tests.

The photograph of the experimental setup is shown in Figure 5a. The DFB laser and the TFLN chip are mounted on independent translation stages, and the laser output is precisely aligned to the bus waveguide; the chip output is collected with a lensed fiber. The split resonances depicted in Figure 4b are pumped, leading to SIL enabled by the pronounced coupling between the counter-propagating modes, which provides the necessary feedback. While the coupling in this instance arises from fabrication imperfections, it can be deterministically induced by incorporating angular gratings within the microresonator [25]. SIL requires an appropriate optical feedback phase [19], which we tune by translating the DFB laser on a piezoelectric stage. With proper phase, scanning the DFB laser frequency across a cavity mode produces characteristic step-like features in the comb power (measured after pump suppression by a notch filter), as shown in Figure 5b. Three low-noise plateaus are observed reproducibly. Setting the drive current within a given plateau enables deterministic access to the corresponding soliton state. Note that the split resonances can increase the parametric oscillation threshold but does not exclude soliton formation in our case [25].

Representative spectra are shown in Figure 5c. The second-highest plateau corresponds to a two-soliton state, which exhibits spectral modulation from soliton interference, while the lowest plateau yields a single-soliton state with a smooth sech^2^ envelope. The comb spans 1,480–1,660 nm, with central lines reaching −7.4 dBm. A dominant comb line is observed, originating from the residual pump transmitting directly through the waveguide. Theoretical considerations indicate that this dominant line would be effectively suppressed if the output were monitored via a dedicated drop port integrated into the device. Excluding the residual pump, the single-soliton pump-to-comb conversion efficiency is 5.2 %. The wall plug efficiency of our DFB-laser–pumped soliton microcomb system is calculated to be approximately 1.1 %, accounting for both the DFB laser’s electrical-to-optical conversion efficiency (20.8 %) and the pump-to-comb conversion efficiency.

A key benefit of SIL is turnkey initiation of the comb [19]: once the current is preset, solitons form automatically upon laser activation, regardless of the laser tuning speed and direction. We emulate repeated start-up by square-wave modulating the drive current (Figure 5d). Each cycle deterministically returns the system to the single-soliton state at the chosen current, certifying the robustness of the turnkey initiation. Additionally, in *z*-cut TFLN, the opposite photothermal and photorefractive responses partially balance each other, creating a thermally stable red-detuned regime [15]. This intrinsic stability, together with SIL’s optical feedback, maintains the laser–cavity detuning within the soliton existence range.

### 3.3 Coherence characterization

**Figure 6:**
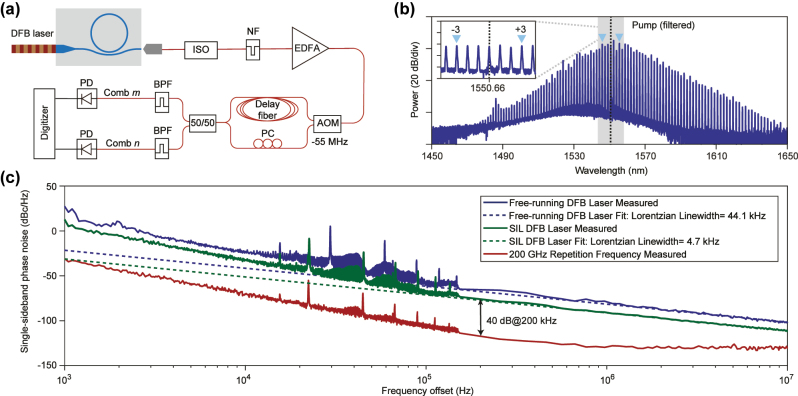
Coherence characterization of soliton microcombs. (a) Measurement setup. ISO: isolator; NF: notch filter; EDFA: erbium-doped fiber amplifier; AOM: acousto-optic modulator; PC: polarization controller; BPF: band-pass filter; PD: photodetector. (b) Soliton spectrum after the NF. The ±3 comb lines used for repetition-rate phase-noise measurement are indicated by triangular markers; the filtered pump is shown by the vertical dashed line. (c) Single-sideband phase noise of the free-running DFB laser, the SIL DFB laser, and the comb repetition frequency.

We assess comb coherence using a delayed self-heterodyne (DSH) interferometer (Figure 6a). The DSH method enables sensitive phase-noise measurement of high-repetition-rate combs by arbitrarily selecting a pair of comb lines [26]. The setup employs a 1 km fiber delay and an AOM driven at 55 MHz by a low-noise source. The undiffracted (zero-order) and first-order AOM outputs form the two interferometer arms, which are recombined and detected. The interference signal is recorded on an oscilloscope; instantaneous frequency fluctuations are extracted via a Hilbert-transform demodulation to obtain the single-sideband phase-noise power spectral density. To measure the phase noise of the soliton repetition frequency, we simultaneously acquire the DSH signals of the ±3 comb lines (Figure 6b) and, after demodulation, compute their differential phase. Using the comb constraint, this directly yields the phase evolution of the repetition frequency [26], [27].

To mitigate etalon ripples from the delay fiber at high offsets ( >200 kHz), the noise is sampled at discrete frequencies *f*_*n*_ = (2*n* − 1)/(2*τ*) with *τ* = 4.8821 μs. Figure 6c compares the phase noise of the DFB laser in free-running and SIL states, together with the 200 GHz comb repetition frequency. The free-running laser exhibits a Lorentzian linewidth of ∼44.1 kHz, which narrows to ∼4.7 kHz under SIL. The measured noise-reduction factor (NRF) is approximately 10 dB, which is 27 dB below the maximum estimated value (see Supplementary Materials). The measurement was conducted under conditions where comb formation occurred, and the associated nonlinear processes likely introduced additional losses that reduced the effective NRF. The comb repetition frequency reaches −130 dBc/Hz at a 1 MHz offset. Because at sub-kilohertz offset frequencies the system is sensitive to environmental perturbations, we focus on the 1 kHz to 10 MHz range to investigate the noise origin. Below ∼200 kHz, the phase-noise spectra of the SIL laser and the repetition frequency display similar features, indicating direct noise transduction from pump to comb. A quantitative transduction factor is obtained at a 200 kHz offset, yielding ∼40 dB from the SIL-laser phase noise to the repetition frequency phase noise.

## 4 Discussion

Future packaging of the demonstrated integrated soliton microcombs will enable robust operation outside laboratory environments [19], [28]. Nevertheless, the full potential emerges when cointegrated with other LN components on the same die. For instance, colocating modulators and comb sources supports massively parallel wavelength-division-multiplexed links from chip-to-chip interconnects to long-haul systems, with the latter benefiting from low-noise amplification in Er-doped TFLN waveguides [29]. Besides, completing comb functionality by adding PPLN waveguides enables *f*–2*f* self-referencing [7].

Progress toward this vision requires (i) migration to X-cut LN to maximize electro-optic and second-harmonic efficiencies [17], [30]; (ii) higher comb line power, e.g., via dark-pulse operation in normal-dispersion microresonators [16], [31]; and (iii) broader span through dispersion engineering [18] and coupled-resonator structures [32], together with low-loss claddings that provide thermal tuning while preserving high optical *Q* [33]. Also, moving to microwave-rate soliton microcombs shall unlock more applications in microwave photonics and precision measurements [7], but device improvements in *Q* and facet coupling efficiency are essential. With continued advances in microfabrication and a deepening understanding of microcomb dynamics [8], fully integrated TFLN comb circuits for communications [34], [35], portable optical clocks [14], and artificial intelligence [36] are within reach.

## Supplementary Material

Supplementary Material Details
