# Supplementary material for: Electrically pumped soliton microcombs on thin-film lithium niobate
Source: Nanophotonics. 2025 Dec 11;14(26):4813–9. doi: 10.1515/nanoph-2025-0510 (PMC12714037; doi:10.1515/nanoph-2025-0510)
Supplement: Supplementary file 1 — Supplementary Material Details [file j_nanoph-2025-0510_suppl_001.pdf]

Supplementary information: Electrically-pumped soliton microcombs on thin-film lithium niobate

Xiaomin Lv[1,2†], Ze Wang[1†], Tianyu Xu[1†], Chen Yang[1†,*], Xing jin[1], Binbin Nie[1], Du Qian[1], Yanwu Liu[1], Kaixuan Zhu[1], Bo Ni[1], Qihuang Gong[1,2,4,5], Fang Bo[3,*], and Qi-Fan Yang[1,2,4,5]
[1]State Key Laboratory for Artificial Microstructure and Mesoscopic Physics and Frontiers Science Center for Nano-optoelectronics, School of Physics, Peking University, Beijing, 100871, China
[2]Hefei National Laboratory, Hefei, 230088, China
[3]Nankai University, Tianjin, 300071, China
[4]Collaborative Innovation Center of Extreme Optics, Shanxi University, Taiyuan, 030006, China
[5]Peking University Yangtze Delta Institute of Optoelectronics, Nantong, 226010, China
[†]These authors contributed equally to this work.
[*]Corresponding author: ycoptics@pku.edu.cn; bofang@nankai.edu.cn;

CONTENTS

I. Theory 2
A. Self-Injection Locking Master Equations 2
B. Noise Reduction Factor 3

II. Comparison of Electrically Pumped Microcomb 3

III. TFLN Waveguide Facet Characterization 4

References 4

I. THEORY

A. Self-Injection Locking Master Equations

We model the self-injection-locked (SIL) soliton microcomb in thin-film lithium niobate (TFLN) microresonator using three fields[1,2]: the soliton optical field, A_S; the backscattered field, A_B; and the laser field, A_L. The full mean-field equations are:

$$\frac{\partial A_S}{\partial T} = -\frac{\kappa}{2}A_S - i\delta\omega_p A_S + i\frac{D_2}{2}\frac{\partial^2 A_S}{\partial \phi^2} + ig_R\big(|A_S|^2 + 2|A_B|^2\big)A_S + i\beta\frac{\kappa}{2}A_B + \sqrt{\kappa_e\kappa_L}\,A_L e^{i\phi_B}, \tag{S1}$$

$$\frac{\partial A_B}{\partial T} = -\frac{\kappa}{2}A_B - i\delta\omega_p A_B + ig_R\left(|A_B|^2 + 2\int_0^{2\pi}\frac{|A_S|^2}{2\pi}d\phi\right)A_B + i\beta\frac{\kappa}{2}\overline{A_S} \tag{S2}$$

$$\frac{\partial A_L}{\partial T} = -\frac{\kappa_L}{2}A_L - i(\delta\omega_p - \delta\omega_L)A_L + \frac{g(|A_L|^2)}{2}(1+i\alpha_g)A_L + \sqrt{\kappa_e\kappa_L}\,e^{i\phi_B}A_B. \tag{S3}$$

Here T is the slow time and ϕ is the angular coordinate in the co-rotating frame. D_2 is the group-velocity dispersion of the resonator. The total cavity loss rate is $\kappa = \kappa_0 + \kappa_e$, with κ_0 and κ_e representing the intrinsic and external coupling loss rates of cavity respectively. $|A_\mathrm{B}|^2$, $|A_\mathrm{L}|^2$, and $\int_0^{2\pi}|A_\mathrm{S}|^2\,d\phi/(2\pi)$ are normalized to intracavity photon numbers, and field amplitude in the pump mode is defined as $\overline{A_\mathrm{S}} = \int_0^{2\pi}A_\mathrm{S},\mathrm{d}\phi/(2\pi)$. The nonlinear coefficient is defined as $g_R = \hbar\omega_0^2 c n_2/(n_0^2 V_\mathrm{eff})$, where V_eff is the effective mode volume, n_0 is the refractive index, and n_2 is the Kerr nonlinear index. The detunings $\delta\omega_p$ and $\delta\omega_L$ denote the frequency offsets of the cold cavity resonance from the SIL and free-running laser frequency, respectively. The parameter β is characterizes the normalized backscattering strength. The pump-laser gain follows $g(|A_L|^2) = g_0/(1+|A_L|^2/|A_{L,\mathrm{sat}}|^2)$. ϕ_B is the propagation phase delay between the resonator and the laser. The total loss rate of the laser mode is dominated by the external coupling κ_L, which permits a simplified analytical treatment.

Because the laser dynamics are much faster than the cavity dynamics, Eq. (S3) may be adiabatically eliminated, yielding the normalized SIL equations[1]:

$$\frac{\partial \psi_S}{\partial \tau} = -(1+i\zeta)\psi_S + id_2\frac{\partial^2\psi_S}{\partial\phi^2} + i(|\psi_S|^2 + 2|\psi_B|^2)\psi_S + i\beta\psi_B + f, \tag{S4}$$

$$\frac{\partial \psi_B}{\partial \tau} = -(1+i\zeta)\psi_B + i(|\psi_B|^2 + 2P)\psi_B + i\beta\overline{\psi_S}, \tag{S5}$$

$$\xi = \Delta_\mathrm{L} + K_0\,\mathrm{Im}\left[(1-i\alpha_g)e^{i2\phi_B}\frac{\psi_B}{f}\right]. \tag{S6}$$

The normalized variables are:

$$\tau = \tfrac{\kappa}{2}T,\quad \psi_S = \sqrt{\tfrac{2g_R}{\kappa}}\,A_S,\quad \psi_B = \sqrt{\tfrac{2g_R}{\kappa}}\,A_B,\quad P = \int_0^{2\pi}\tfrac{|\psi_S|^2}{2\pi}\,d\phi,\quad \zeta = \tfrac{2\delta\omega_p}{\kappa},$$

$$\Delta_L = \tfrac{2\delta\omega_L + \kappa_L\alpha_g}{\kappa},\quad d_2 = \tfrac{D_2}{\kappa},\quad f = \tfrac{2\sqrt{\kappa_e\kappa_L}}{\kappa}\sqrt{\tfrac{2g_R}{\kappa}}\,A_L e^{i\phi_B},\quad K_0 = \tfrac{4\kappa_e\kappa_L}{\kappa^2}.$$

In this normalization, $|f|^2$ corresponds to the pump power in units of the parametric-oscillation threshold of the standalone resonator. Eqs. (S4)–(S6) are solved using a split-step Fourier method. Unless stated otherwise, simulation parameters use:

$$\alpha_g = 0,\qquad |f|^2 = 4,\qquad \beta = 1.2,\qquad K_0 = 50,\qquad d_2 = 0.034.$$

Figure S1 summarizes representative SIL soliton-formation behavior in TFLN microresonator.

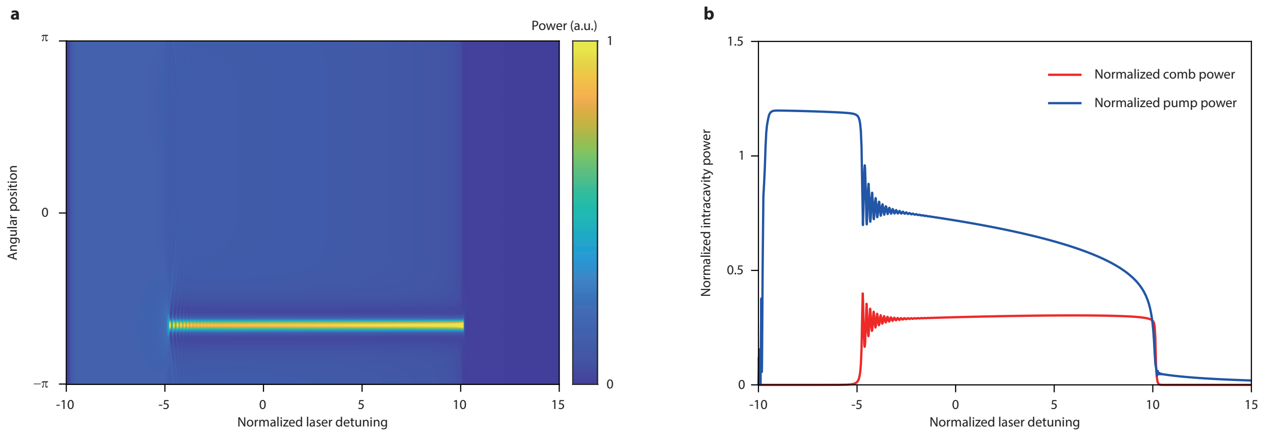

Fig. S1. **Numerical simulations of SIL soliton generation in TFLN microresonator.** (a) Spatiotemporal evolution of the intracavity field versus normalized laser detuning, showing deterministic soliton initiation and stable propagation . (b) Corresponding intracavity power (red: comb power, blue: pump power) as functions of normalized laser detuning.

B. Noise Reduction Factor

The noise-reduction performance of a SIL laser is determined by the spectral response and strength of the backscattered field, as described by the full theory that accounts for both the laser and microresonator dynamics[3]. In the regime of strong backscattering—where the cavity mode splits into a resolvable doublet—the noise-reduction factor (NRF) approaches the saturated value:

$$\mathrm{NRF} \approx 4(1+\alpha^2)\cdot T^2\cdot \eta^2 \cdot \left(\frac{Q_R^2}{Q_d^2}\right) \tag{S1}$$

Here, Q_R refers to the Q value of the microresonator quality factor, whereas Q_d characterizes the Q value of the laser diode. The resonator loading factor is given by $\eta = Q_R/Q_e$, where Q_e denotes the external coupling quality factor associated with power transfer between the bus waveguide and the resonator. The term T quantifies the insertion loss from the DFB laser facet to the TFLN waveguide, and α represents the amplitude–phase coupling coefficient of the laser. In our device, representative values are $\alpha = 2.5$, $T = -7$ dB, $\eta = 1/3$, and $Q_d = 10^4$. Using these parameters, a cavity mode with a loaded quality factor of 2×10^6 and observable modal splitting yields a maximum noise-reduction factor of approximately 37 dB.

II. COMPARISON OF ELECTRICALLY PUMPED MICROCOMB

We make a summary of representative electrically pumped microcombs across different material platforms.

TABLE I. Comparison of electrically pumped microcomb platforms.

Platform	Q-factor	Laser–chip Coupling	FSR	Pump Type	Physical mechanism	Pump-to-comb efficiency	WPE of laser/system
**TFLN**	**3×10^6**	**7 dB**	**200 GHz**	**Electrically pumped**	**Bright soliton**	**5.2%**	**20.8% / 1.1%**
TFLN[4]	–	3.4–3.9 dB	10.17 GHz	Electrically pumped	Active MLL	–	–
TFLN[5]	2.7×10^6	–	200 GHz	Electrically pumped	Bright soliton	–	–/1.1%
SiN[1]	16×10^6	3–5 dB	15–40 GHz	Electrically pumped	Bright soliton	–	20% / –
SiN[6]	10×10^6	–	26.2 GHz	Electrically pumped	Platicon	40%	–
SiN[7]	–	–	300 GHz	Electrically pumped	Kerr comb	12.5%	–
SiN[8]	–	0.4 dB	3 GHz	Electrically pumped	Passive MLL	–	–
SiN[9]	3×10^6	–	198-792 GHz	Electrically pumped	Kerr comb	–	30%/–
SiN[10]	25×10^6	6.4 dB	10.7 GHz	Electrically pumped	Platicon	8%	–
AlN[11]	2.9×10^6	5 dB	230 GHz	Electrically pumped	Platicon	–	–
Germano-silicate[12]	303×10^6	–	–	Electrically pumped	Bright soliton	–	–

Note: MLL denotes a mode-locked laser.

III. TFLN WAVEGUIDE FACET CHARACTERIZATION

To evaluate the DFB laser-to-chip coupling efficiency, we performed scanning electron microscope (SEM) imaging of the diced facet of the TFLN waveguide. Figure S1 shows a representative SEM image acquired at 22.47 kX magnification. The facet exhibits significant surface topography variations.

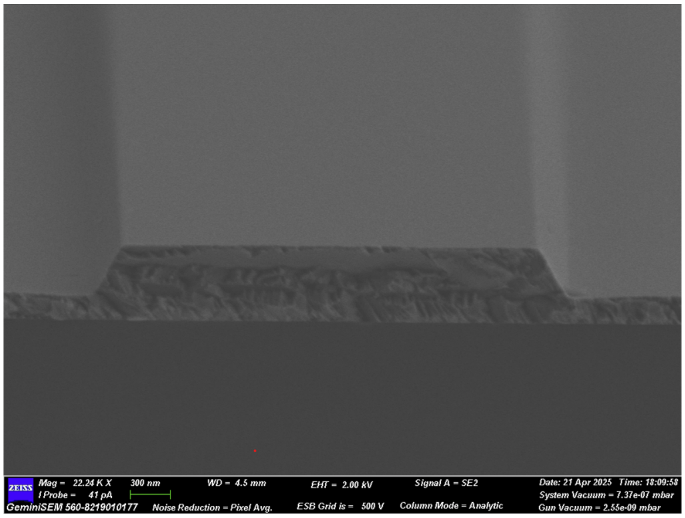

Fig. S2. SEM image of the diced TFLN waveguide facet.

[1] Shen, B. *et al.* Integrated turnkey soliton microcombs. *Nature* **582**, 365–369 (2020).
[2] Wang, Z. *et al.* Integrated soliton microcombs beyond the turnkey limit. *arXiv preprint arXiv:2511.06909* (2025).
[3] Jin, W. *et al.* Hertz-linewidth semiconductor lasers using cmos-ready ultra-high-Q microresonators. *Nat. Photon.* **15**, 346–353 (2021).
[4] Guo, Q. *et al.* Ultrafast mode-locked laser in nanophotonic lithium niobate. *Science* **382**, 708–713 (2023).
[5] Hu, Q. *et al.* Chip-scale ultrafast soliton laser. *arXiv preprint arXiv:2510.15146* (2025).
[6] Lihachev, G. *et al.* Platicon microcomb generation using laser self-injection locking. *Nat. Commun.* **13**, 1771 (2022).
[7] Ulanov, A. E. *et al.* Synthetic reflection self-injection-locked microcombs. *Nat. Photonics* **18**, 294–299 (2024).
[8] Hermans, A. *et al.* High-pulse-energy iii-v-on-silicon-nitride mode-locked laser. *APL Photonics* **6**, 096102 (2021).
[9] Gil-Molina, A. *et al.* High-power electrically pumped microcombs. *Nat. Photonics* **19**, 1270–1274 (2025).
[10] Sun, W. *et al.* A chip-integrated comb-based microwave oscillator. *Light Sci. Appl.* **14**, 179 (2025).
[11] Ding, Y. *et al.* Self-injection locking dynamics with raman actions in aluminum nitride microresonators. *Phys. Rev. Lett.* **135**, 093801 (2025).
[12] Chen, H.-J. *et al.* Germano-silicate ultra-low loss photonic integrated circuits across visible and near-infrared spectrum. In *2024 Conference on Lasers and Electro-Optics (CLEO)*, 1–2 (2024).
